# Gonadocorticoids Have Different Effects on the Expression of Toll-like Receptors When Infected with Various HIV-1 Subtypes

**DOI:** 10.3390/v17111512

**Published:** 2025-11-18

**Authors:** Marina Nosik, Konstantin Ryzhov, Elena Berezhnaya, Elizaveta Bystritskaya, Olga Lobach, Irina Kiseleva, Elizaveta Kostyuchenko, Anna Kuzina, Ekaterina Meremianina, Dmitry Kireev, Oxana Svitich

**Affiliations:** 1 I.I. Mechnikov Institute of Vaccine and Sera, 105064 Moscow, Russia; rkazaw@yahoo.com (K.R.); elvenel@gmail.com (E.B.); lisabystritskaya@gmail.com (E.B.); victoriola@yandex.ru (O.L.); iakiseleva@yandex.ru (I.K.); pontigruel@gmail.com (E.K.); kuzvitanna@yandex.ru (A.K.); ekaterina@meremianina.ru (E.M.); svitichoa@yandex.ru (O.S.); 2 Department of Microbiology, I.M. Sechenov First Moscow State Medical University, 119048 Moscow, Russia; 3 Department of Virology, I.M. Sechenov First Moscow State Medical University, 119048 Moscow, Russia; 4 Central Research Institute of Epidemiology, 111123 Moscow, Russia; dmitkireev@yandex.ru; 5 Department of Immunology, I.M. Sechenov First Moscow State Medical University, 119048 Moscow, Russia

**Keywords:** estradiol, progesterone, HIV-1 subtype B, HIV-1 sub-subtype A6, TLR2, TLR4, TLR9

## Abstract

Recent studies suggest that immune response to pathogens may vary depending on changes in hormone levels. Toll-like receptors (TLRs) are the key components of the innate immune system and play a crucial role in HIV infection. Given the significant genetic diversity of HIV-1, this study examined the effect of female sex hormones on the several TLR2, TLR4, and TLR9 expression in human peripheral blood mononuclear cells (PBLs) isolated from different female donors and infected with different variants of HIV-1 subtypes A6 and B. Thus, high doses of hormones upregulated the TLR2 and TLR9 expression in PBLs infected only with v1.A6, which also correlated with an increased viral load: by 3.8 times (*p* = 0.0033) when cells were treated with estradiol and by 4.4 times (*p* = 0.006) when treated with progesterone. Hormones did not modulate TLRs expression in the cells infected with subtype B, with the exception of one donor. In PBLs from this donor infected with the v1.B variant, hormones upregulated TLRs expression, which also correlated with the increased viral load (1.3-fold increase (*p* = 0.0036)). Hence, it was shown that gonadal steroids can play an important role in HIV-1 replication and immune response to a pathogen. Moreover, it was shown that different isolates of the same subtype may have distinct biological properties. The detected diversity in the TLRs expression in infected PBLs from different donors indicates that host genetics may also play an important role in HIV susceptibility.

## 1. Introduction

HIV infection, along with other diseases, remains a serious global health issue. In 2024, the total number of people living with HIV worldwide was estimated at 40.8 million [1]. Thanks to antiretroviral therapy (ART), people living with HIV have been able to significantly extend their lives and improve their quality of life [2,3]. However, in 2024, over 600,000 people died from AIDS-related diseases [1]. One of the reasons for this is the fact that HIV infection is characterized by chronic immune activation, which persists to some extent even with antiretroviral therapy [4,5,6]. HIV-1, through complex mechanisms of interaction with the body’s immune system (particularly through interaction with Toll-like receptors (TLRs)), disrupts the immune response, leading to abnormal immune activation and inflammation [4].

It is known that TLRs are immune factors of the first line of defense, mainly due to their recognition of pathogen-associated molecular patterns (PAMPs) [7,8,9,10]. As key pathogen-sensing receptors, they respond to various antimicrobial ligands and trigger both innate and adaptive immune responses to infection [11]. Currently, there is a significant body of evidence suggesting that TLRs, in particular, play a crucial role in the pathogenesis of HIV infection [7,10,11,12]. First of all, TLRs can directly interact with already infected cells and activate signaling pathways that regulate HIV replication [11,12]. At the same time, cells that are not fully permissive to infection, already at the stage of HIV entry into the cell, can also participate in TLR-dependent immunity, leading to the release of cytokines and the initiation of cell-mediated responses that affect viral replication [11]. Defective viral particles that are unable to fully infect but are significantly more abundant than infectious particles may also play an important role in activating the TLR-dependent response in HIV infection [11,13]. However, excessive activation of TLRs can destabilize immune homeostasis and lead to the development of inflammatory and autoimmune pathologies [9].

It should be noted that gender differences are an important physiological factor that determines the body’s immune response to various pathogens. Sex hormones and sex chromosomes, along with epigenetic factors, regulate the expression of receptors and signaling pathways, affecting the progression and outcome of the disease [14]. Many immune genes are located on the X chromosome, including certain TLRs that recognize single-stranded viral RNA (ssRNA), including HIV genomic RNA [11,15,16,17]. It has been shown that although women have a lower viral load (VL) than men when infected with HIV-1, they progress to AIDS at the same rate as men, and in some cases even faster [18,19,20,21,22,23]. This may be due to the fact that women living with HIV have higher levels of immune activation and interferon-stimulated gene expression, which is mediated by gender-specific responses in plasmacytoid dendritic cells (pDCs) [18,19,21,23].

An increasing number of studies suggest that immune responses to pathogens may vary depending on changes in hormone levels, which naturally fluctuate throughout the menstrual cycle, during pregnancy, and when using contraceptives [18,24,25]. It has been suggested that susceptibility to HIV-1 increases during the so-called “vulnerable period” in the second half of the menstrual cycle after ovulation [24,26]. During ovulation, estrogen levels reach their peak, which in some cases can lead to increased replication of HIV-1, increasing the viral load [19,26].

Steroid hormones can directly affect HIV-1 replication by modulating the expression of chemokine receptors and TLRs, but their complex effects can be variable and depend on the hormone concentrations [15,27,28,29,30,31,32]. Previously, our group showed that female sex hormones can modulate the expression of TLRs to various degrees when infected with different subtypes of HIV-1 (one variant of each subtype was used in that study) [33]. To date, there is evidence that significant divergences in regulatory genes and long terminal repeat (LTR) regions can be found between different isolates of the same HIV-1 subtype [28,29,33,34,35]. Therefore, given the significant genetic diversity of HIV-1 both between subtypes and within a single subtype, this study examined the effect of female sex hormones on the expression of several TLRs (TLR2, TLR4, and TLR9) in human peripheral blood mononuclear cells (PBLs) isolated from different female donors and infected with various variants of HIV-1 subtypes A6 and B.

## 2. Materials and Methods

### 2.1. Peripheral Blood Mononuclear Cells (PBLs)

Human peripheral blood mononuclear cells (PBMCs) were isolated by standard Ficoll density gradient centrifugation from the blood of 9 HIV-1 seronegative female donors (initially there were 12 donors but 3 were excluded from this study afterwards). The female donors were of 25–30 years and at the time of blood collection, none were using exogenous hormones.

### 2.2. Ethical Aspects

Written informed consent was obtained from volunteer healthy donors (>18 years old) prior to blood collection according to the ethical standards of the international ethical guidelines in the field of biomedical research with human participation. This study was conducted according to the guidelines of the Declaration of Helsinki and approved by the Bio-medical Ethics Committee of the I.I. Mechnikov Institute of Vaccines and Sera, Moscow, Russia (#2/03/11/20).

### 2.3. HIV-Infection

PBMCs were stimulated with phytohemagglutinin (PHA) and then infected with different HIV-1 subtypes (0.001 TCID50/cell): A6, v1.A6 (GenBank: BankIt2701146 VSMO70 OQ979187); v2.A6 (BankIt2701146 VSMO71 OQ979188); B, v1.B (BankIt2701146 IM735 OQ979192); v2.B (BankIt2701146 VSMO78 OQ979189) from the I.I. Mechnikov Institute of Vaccines and Sera HIV-1 isolates panel. The HIV-1 isolates used in this study had different HIV-1 drug resistance profiles (Supplementary File Table S1): OQ979187 sequence has a K103N mutation in the reverse transcriptase. Due to this mutation, the virus has a high level of resistance to NNRTIs nevirapine and efavirenz; the K103N mutation has almost no effect on the fitness of the virus/OQ979188 sequence has an E138A mutation in the reverse transcriptase. Due to this mutation, the virus has a low level of resistance to the NNRTIs dopavirine and rilpivirine, as well as a potentially low level of resistance to etravirine; the E138A mutation slightly reduces the fitness of the virus/OQ979192 sequence has a V179D mutation in the reverse transcriptase. Due to this mutation, the virus has a potentially low level of resistance to NNRTI drugs such as dapivirine, efavirenz, etravirine, nevirapine, and rilpivirine; the V179D mutation significantly reduces the viral replicative activity/OQ979189 sequence does not have any resistance mutations. The isolates also differed in their tropism: v1.B/OQ979192 was CXCR4-tropic, while the other three HIV-1 isolates were CCR5-tropic: v1.A6/OQ979187; v2.A6/OQ979188; and v2.B/OQ979189 (Supplementary File Table S2).

After 2 h of PBMCs incubation with the virus at 37 °C in 5% CO_2_, the PBMCs were washed twice with 1× phosphate-buffered saline (PBS). PBLs were cultured for 7 days in RPMI-1640 medium supplemented with 15% FBS (Sigma-Aldrich, Darmstadt, Germany, F9665), 100 U/mL of interleukin-2 (Sigma-Aldrich, Darmstadt, Germany, H7041), 2 mM glutamine, 100 U/mL of penicillin, and 100 U/mL of streptomycin. Three independent experiments with the PBMCs of each donor was performed (the blood samples were taken from each donor 3 times after a certain time) and each experiment was run in triplicate. The HIV replication pattern did not vary significantly and was comparable between donor’s PBMCs. The PBMCs from donors (3 donors) which were not successfully infected were excluded from this study.

### 2.4. Hormone Treatment

PBMCs were treated with various physiological concentrations either of 17β-estradiol: 0.04 nM, 100 nM (Sigma, St. Louis, MO, USA, E2257) or progesterone: 3.2 nM, 64 nM (Sigma-Aldrich, Darmstadt, Germany, P7556). In order to study the effect of the hormones during the exponential phase of the virus replication, a scheme of the simultaneous introduction of the hormones and virus was chosen.

### 2.5. Assessment of Virus Replication

Viral load in samples of cellular supernatants was determined using RT-qPCR on the 7th day post infection. This study was performed from 100 μL of supernatant using the Amplisens HIV-monitor-FRT reagent kit (CRIE, Moscow, Russia) as per the manufacturer’s instructions. According to the manufacturer, quantitative HIV RNA determination is performed by amplifying two regions of the HIV-1 genome (5′-LTR and reverse transcriptase gene) and detecting them in real time. In our study, we used 100 µL of supernatant for RNA extraction, so the lower limit of the linear range and analysis sensitivity were 500 HIV RNA copies/mL.

### 2.6. Assessment of Toll-like Receptor (TLR) and C-C Chemokine Receptor (CCR) Gene Expression

To evaluate *TLR2*, *TLR4*, *TLR9*, *CCR5* and *CCR8* gene expression levels real-time reverse transcriptase-polymerase chain reaction (rRT-PCR) was performed. Initially, RNA was extracted from each sample of PBLs with the use of “ExtractRNA” reagent according to the instruction (“Evrogen”, Moscow, Russia). “SYBR Green I” kit (“Syntol”, Moscow, Russia) was used to perform rRT-PCR. The specific oligonucleotides were developed in the laboratory of molecular immunology (Mechnikov Institute of Vaccines and Sera) and then synthesized (“Syntol”, Moscow, Russia): for TLR2, CCA-GCA-AAT-TAC-CTG-TGT-GA (forward primer) and CCC-ACA-TCA-TTT-TCA-TAT-AC (reverse primer); for TLR4, TTC-CCT-GGT-GAG-TGT-GAC-TA (forward primer) and CAC-CTT-TGT-TGG-AAG-TGA-AA (reverse primer); for TLR9, TGG-TGT-TGA-AGG-ACA-GTT-CTC-TC (forward primer) and CAC-TCG-GAG-GTT-TCC-CAG-C (reverse primer); for CCR5, CTG-GCC-ATC-TCT-GAC-CTG-TTT-TTC (forward primer) and CAG-CCC-TGT-GCC-TCT-TCT-TCT-CAT (reverse primer); for CCR8, TGG-CTG-TTG-TCC-ATG-CCG-TGT-A (forward primer) and TGG-GAT-GGT-AGC-CAT-AAT-GGC-G (reverse primer). The following protocol for amplification program was used: incubation at 95 °C for 5 min (1 cycle); denaturation at 95 °C for 15 s, annealing and elongation at 60 °C for 50 s (40 cycles). TLRs expression determination was performed on Day 7 post infection. The 2^(−∆∆C(T))^ method was used for data analysis. *ACTB* gene was used as the reference in order to reveal the relative gene expression level in each individual. In addition, all the values for each case were calculated against the untreated PBLs of each donor.

### 2.7. Statistical Analysis

Statistical analysis and data visualization were performed using SPSS Statistics 17.0 (USA) and GraphPad Prism v9.5.0 (GraphPad Software, Boston, MA, USA). The nonparametric Kruskal–Wallis H-test was used to test the significance of the differences. A *p*-value of <0.05 (*) was considered significant; *p* < 0.01 (**) and *p* < 0.0001 (***) very significant.

## 3. Results

**Figure 1:**
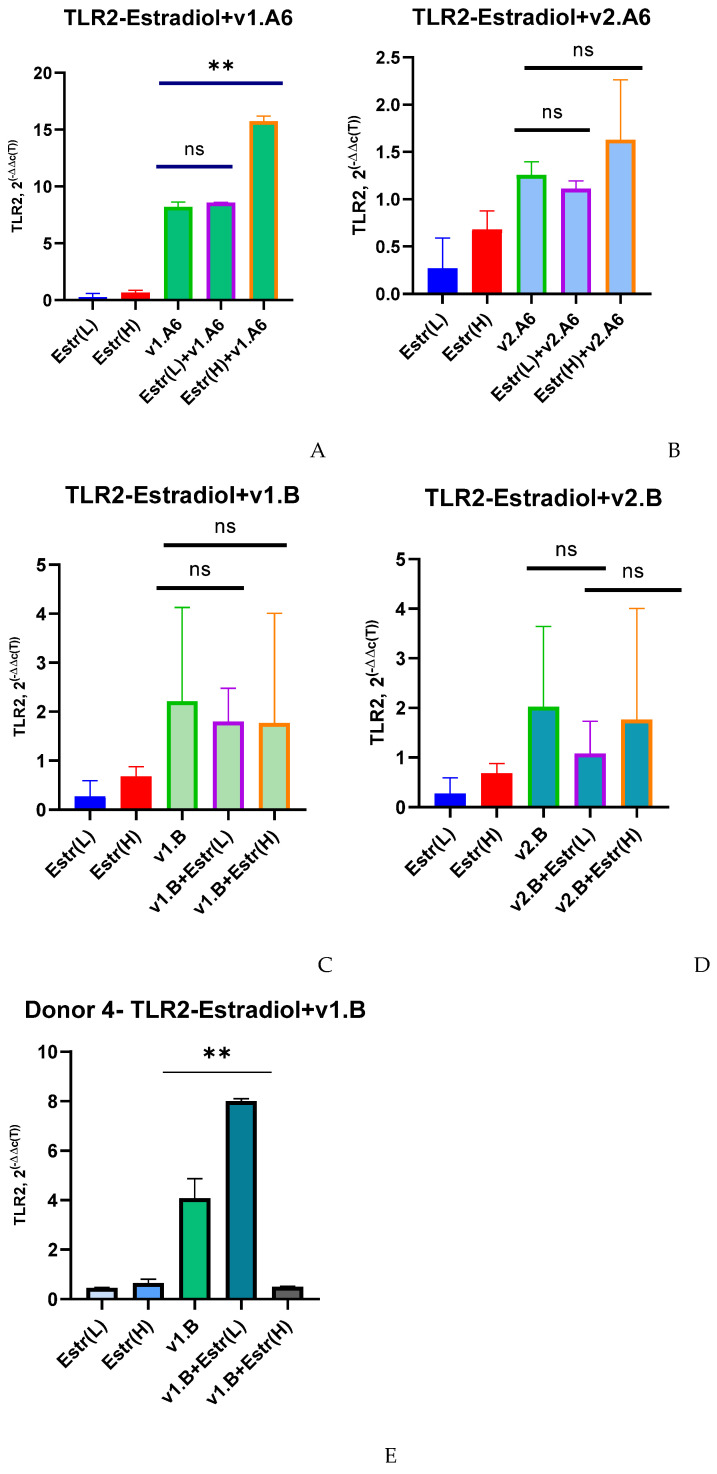
Median values of TLR2 expression in PBMCs of female donors infected with various variants of HIV-1 sub-subtype A6 and subtype B in the presence of (L) low (0.04 nM) and (H) high (100 nM) doses of estradiol (Estr): (**A**) PBMCs +A6, v1.A6; (**B**) PBMCs +A6, v2.A6; (**C**) PBMCs +B, v1.B6; (**D**) PBMCs +B, v2.B6; (**E**) PBMCs Donor 4+ B, v1.B6. PBMCs, peripheral blood lymphocyte; A, HIV-1 sub-subtype A6; B, HIV-1 subtype B; 2^(−∆∆C(T))^, normalized expression coefficient. The median values of each of the three experiments were used to present the statistical analysis (n = 3). ** *p* < 0.001 very significant; ns, no statistical difference, *p* > 0.05.

When PBMCs were infected with HIV-1 sub-subtype A6, v1.A6, it was found that a high dose of estradiol, unlike a low dose, increased TLR2 expression by 1.9 times (*p* = 0.002) compared to infected cells that were not treated with the hormone (Figure 1A). A slightly different pattern was observed when cells were infected with sub-subtype A6, variant v2.A6, and subtype B, variants v1.B and v2.B (except for one donor). In this case, both low and high concentrations of estradiol did not affect the expression of the TLR2 gene (Figure 1B–D).

The exception to this concept was Donor 4. When Donor 4′s PBMCs were infected with the v1.B variant, a 1.96-fold increase in TLR2 gene expression was observed at a low dose of estradiol (*p* = 0.0022) compared to infected PBMCs in the absence of the hormone (Figure 1E).

**Figure 2:**
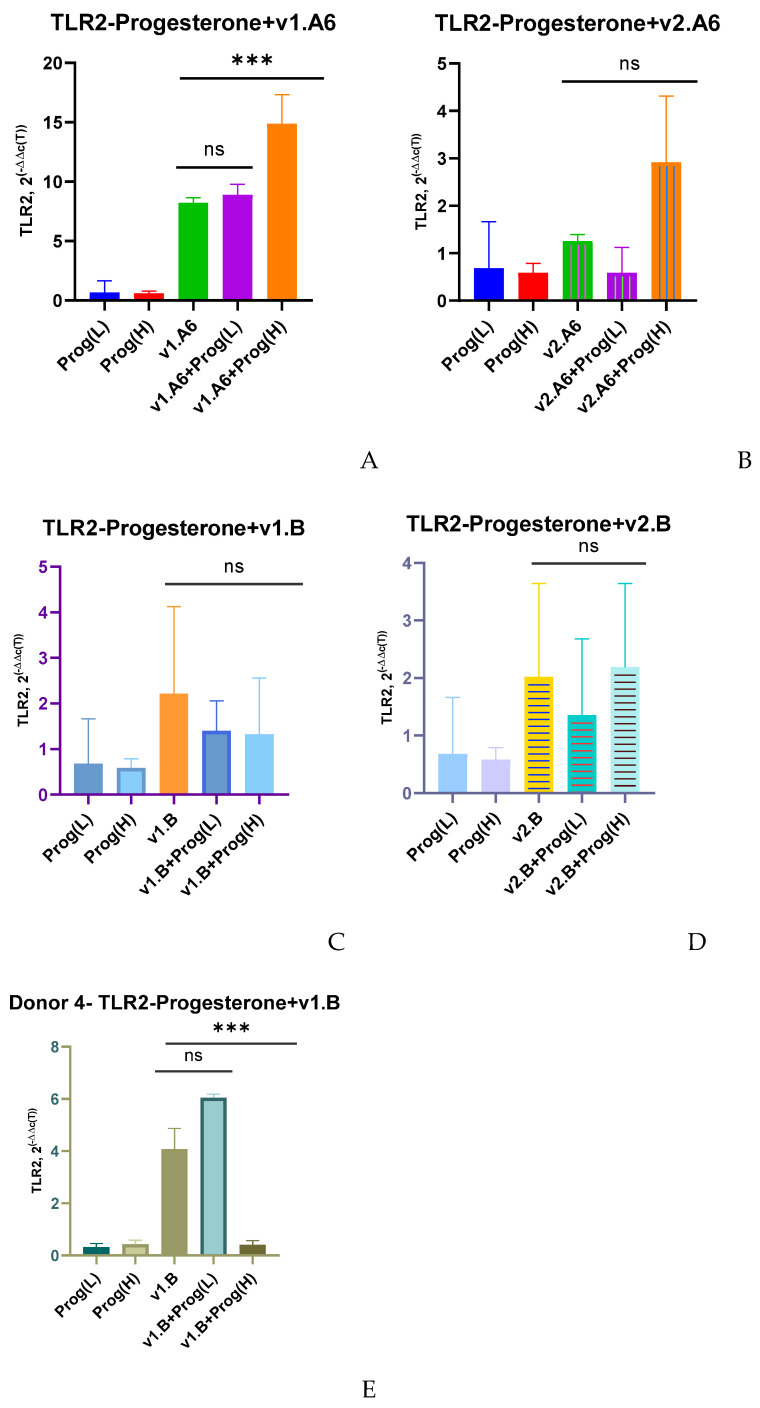
Median values of TLR2 expression in PBMCs of female donors infected with various variants of HIV-1 sub-subtype A6 and subtype B in the presence of (L) low (3.2 nM) and (H) high (64 nM) doses of progesterone (Prog): (**A**) PBMCs +A6, v1.A6; (**B**) PBMCs +A6, v2.A6; (**C**) PBMCs +B, v1.B6; (**D**) PBMCs +B, v2.B6; (**E**) PBMCs Donor 4+ B, v1.B6. PBMCs, peripheral blood lymphocyte; A, HIV-1 sub-subtype A6; B, HIV-1 subtype B; 2^(−∆∆C(T))^, normalized expression coefficient. The median values of each of the three experiments were used to present the statistical analysis (n = 3). *** *p* < 0.0001 very significant, no statistical difference, *p* > 0.05.

In PBMCs infected with variant v1.A6, a high dose of progesterone, as well as in case with estradiol, increased TLR2 gene expression by 1.8 times (*p* = 0.0019) compared with infected PBLs without hormone treatment (Figure 2A). Meanwhile, in the presence of both doses of progesterone in PBMCs infected with two variants of HIV-1 subtype B and the variant of sub-subtype A6, v2.A6, no changes in TLR2 expression were observed (the exception was also Donor 4) (Figure 2B–D).

In the PBMCs of Donor 4 infected with variant v1.B, a 1.5-fold increase in TLR2 gene expression was observed at a low dose of progesterone (*p* = 0.0286) compared to the infected PBLs in the absence of the hormone (Figure 2E).

Low and high doses of both estradiol and progesterone did not have a statistically significant effect on the expression of the TLR4 gene in PBLs infected with two variants of subtype B (v1.B and v2.B) and sub-subtype A6 (v1.A6 and v2.A6) (Supplementary File Figure S1).

**Figure 3:**
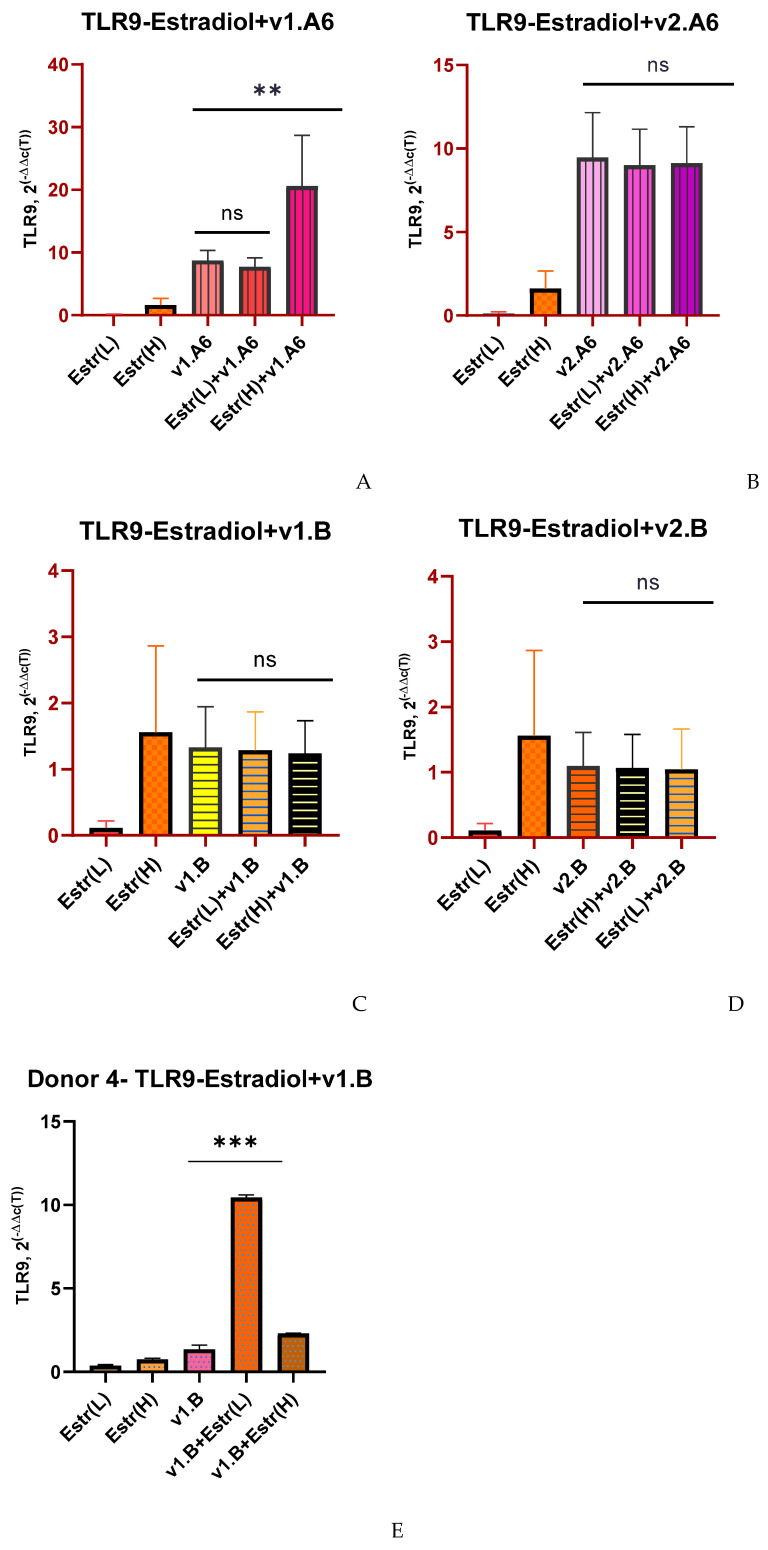
Median values of TLR9 expression in PBMCs of female donors infected with various variants of HIV-1 sub-subtype A6 and subtype B in the presence of (L) low (0.04 nM) and (H) high (100 nM) doses of estradiol (Estr): (**A**) PBMCs +A6, v1.A6; (**B**) PBMCs +A6, v2.A6; (**C**) PBMCs +B, v1.B6; (**D**) PBMCs +B, v2.B6; (**E**) PBMCs Donor 4+ B, v1.B6. PBMCs, peripheral blood lymphocyte; A, HIV-1 sub-subtype A6; B, HIV-1 subtype B; 2^(−∆∆C(T))^, normalized expression coefficient. The median values of each of the three experiments were used to present the statistical analysis (n = 3). *** *p* < 0.0001 and ** *p* < 0.001 very significant; ns, no statistical difference, *p* > 0.05.

Low and high doses of estradiol also had no significant effect on TLR9 expression when PBMCs were infected with two variants of subtype B (v1.B and v2.B) (except for Donor 4) and sub-subtype A6 (v.2A6) (Figure 3B–D). When Donor 4’s PBMCs was infected with the v1.B variant, a 7.8-fold increase in TLR9 gene expression was observed at a low dose of estradiol (*p* = 0.0153) compared to infected PBMCs in the absence of hormone (Figure 3E). As well, the expression of the TLR9 gene increased 2.4-fold in PBMCs infected with v.1A6 in the presence of a high dose of estradiol (*p* = 0.0034) (Figure 3A).

**Figure 4:**
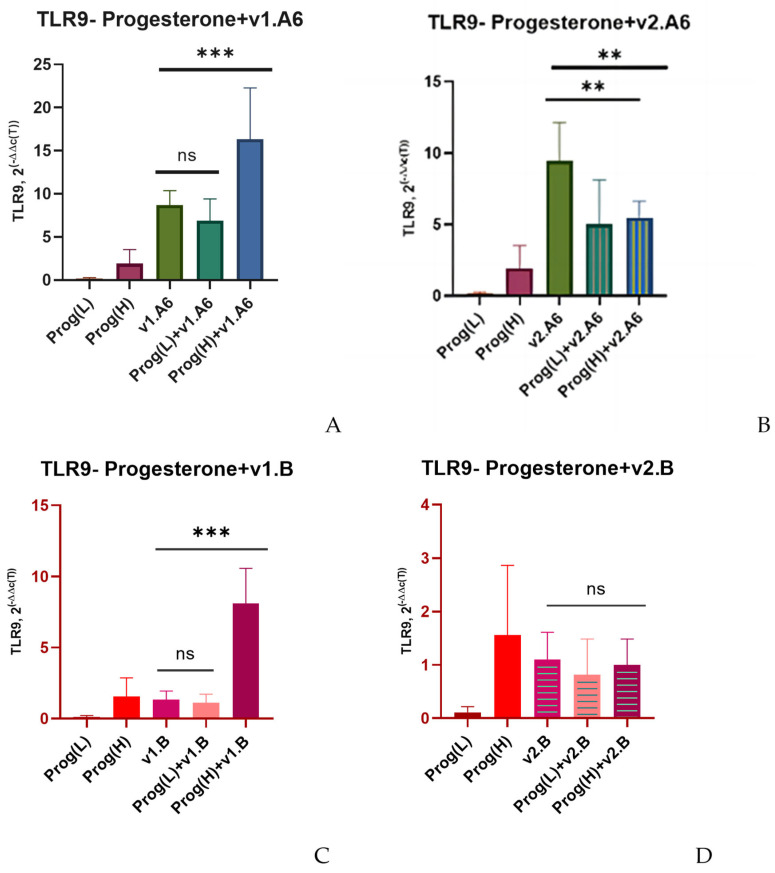

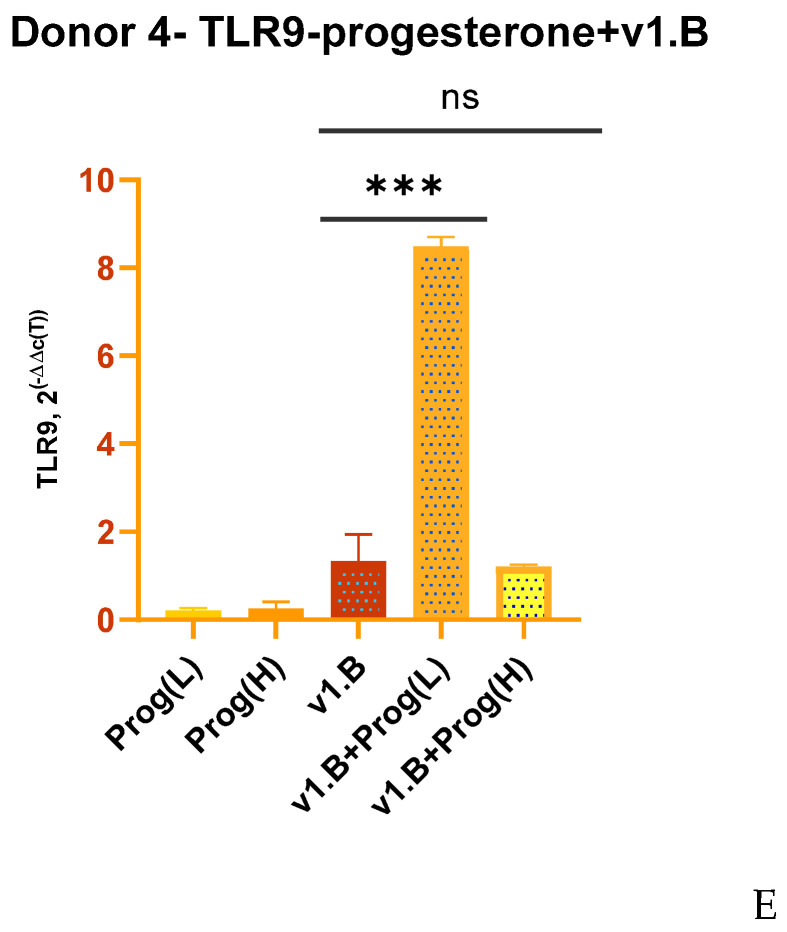
Median values of TLR9 expression in PBMCs of female donors infected with various variants of HIV-1 sub-subtype A6 and subtype B in the presence of (L) low (3.2 nM) and (H) high (64 nM) doses of progesterone (Prog): (**A**) PBMCs +A6, v1.A6; (**B**) PBMCs +A6, v2.A6; (**C**) PBMCs +B, v1.B6; (**D**) PBMCs +B, v2.B6; (**E**) PBMCs Donor 4+ B, v1.B6. PBMCs, peripheral blood lymphocyte; A, HIV-1 sub-subtype A6; B, HIV-1 subtype B; 2^(−∆∆C(T))^, normalized expression coefficient. The median values of each of the three experiments were used to present the statistical analysis (n = 3). *** *p* < 0.0001 and ** *p* < 0.001 very significant; ns, no statistical difference, *p* > 0.05.

In the case of infection with the v1.A6 variant, modulation in TLR9 expression was also observed when PBMCs were treated with a high dose of progesterone: receptor expression increased by 1.9 times (*p* = 0.00331) when cells were infected with v1.A6 (Figure 4A).

Both low and high doses of progesterone, as well as estradiol, did not affect TLR9 expression when PBLs were infected with subtype B variants (v1.B and v2.B) and sub-subtype A6 variant, v.2A6 (Figure 4B–D). The exception, as in the case of estradiol, was Donor 4. In the cells of this donor infected with the v1.B variant, low concentrations of progesterone resulted in a 6.4-fold increase in TLR9 gene expression (*p* = 0.0112) (Figure 4E).

**Figure 5:**
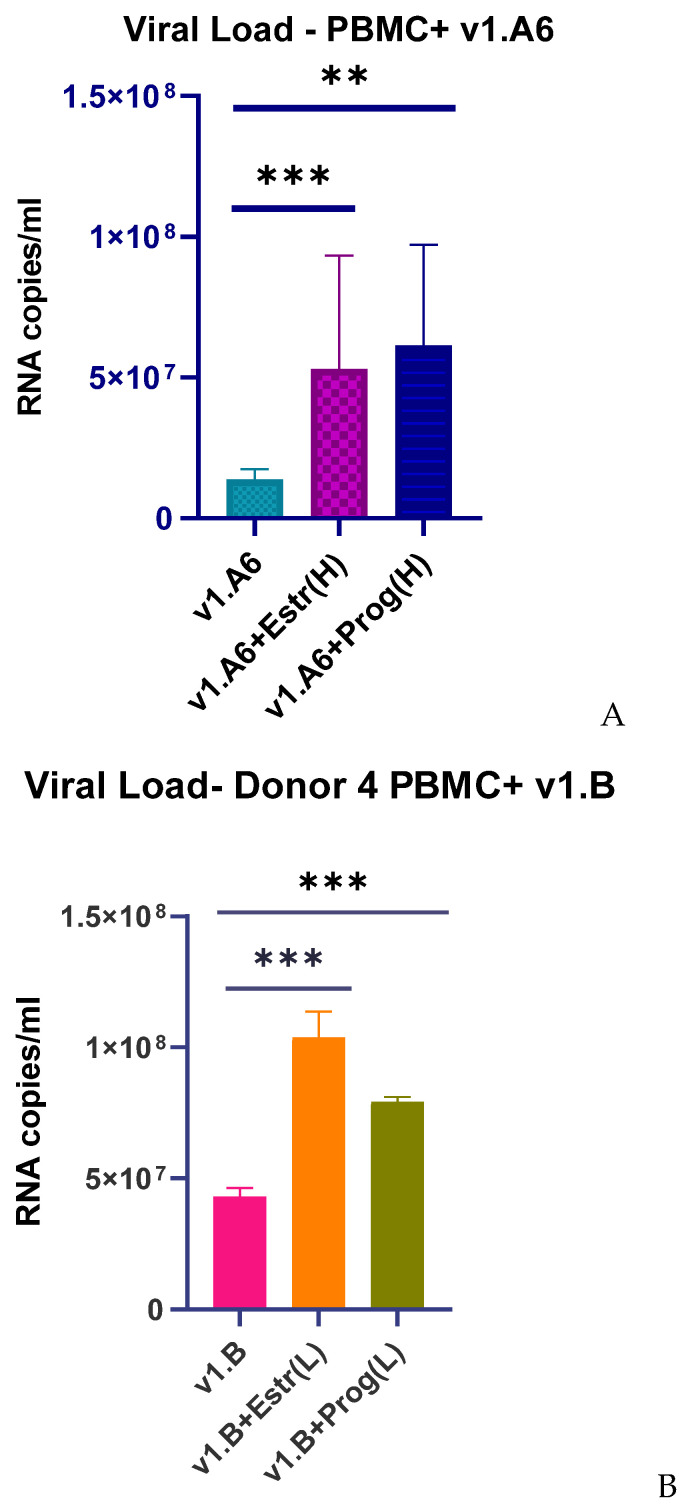
Median values of RNA copies in PBMCs of female donors infected with HIV-1 sub-subtype A6, variant v1.A6 and subtype B, variant v1.B in the presence of (H) high dose (100 nM) of estradiol (Estr) and (L) low dose (3.2 nM) of progesterone (Prog): (**A**) PBMCs + A6, v1.A6 +Estradiol (H); (**B**) PBMCs +B, v1.B6 +Progesterone (L). PBMCs, peripheral blood lymphocyte. The median values of each of the three experiments were used to present the statistical analysis (n = 3). *** *p* < 0.0001 and ** *p* < 0.001 very significant.

Increased expression of TLRs genes in PBLs infected with sub-subtype A6, v1.A6, correlated with a 3.8-fold increase in VL when cells were treated with estradiol (*p* = 0.0033), and a 4.4-fold increase when cells were treated with progesterone (*p* = 0.006) (Figure 5A,B). When Donor 4′s PBMCs were infected with subtype B, variant v1.B, in the presence of estradiol, the VL increased by 1.3 times (*p* = 0.0036). In the presence of progesterone, the VL increased by 1.2 times (*p* = 0.0045).

In order to test whether high doses of gonadosteroids also induce increased expression of co-receptors playing a significant role in HIV-1 infection, we studied co-receptors expression (in particular, CCR5, and CCR8) in uninfected donor’s PBMCs after treatment with high doses of hormones, post infection and in infected PBMCs treated with hormones (Supplementary File Figure S2). There was no statistical difference in CCR5 expression in cells infected whether with sub-subtype A6 variants or B subtype variants, in presence or absence of both hormones. The same was true for CCR8. Though estradiol upregulated CCR8 expression in cells infected with both variants of sub-subtype A6 (*p* = 0.0164 and *p* = 0.0197), there was no statistical difference in co-receptor’s expression between infected PBMCs treated or untreated with hormone. Progesterone also, did not induce CCR8 expression in PBMCs treated with gonadal steroids. In cells infected with the v2.A6 variant, progesterone even downregulated co-receptor’s expression (*p* = 0.0184) compared with infected PBMCs without hormone.

## 4. Discussion

The results of this study showed significant differences in the expression of TLRs and the replication of HIV-1 subtypes A6 and B in infected PBMCs of female donors in the presence of gonadocorticoids, both between different variants of these two subtypes and between variants within the same subtype. High doses of hormones increased the expression of TLR2 and TLR9 in PBLs infected with the HIV-1 A6 sub-subtype, v1.A6, but not with the v2.A6 variant. At the same time, gonadal steroids did not modulate the expression of TLRs in cells infected with both variants of the B subtype, with the exception of one donor. In the PBLs of this donor, infected with the HIV-1 B subtype, v1.B, hormones increased the expression of similar TLRs. In all cases, increased TLRs expression correlated with increased VL.

It is well known that estradiol and progesterone, which are key female sex hormones, have a significant impact on reproductive and non-reproductive aspects of health, controlling a wide range of functions in the female body, including the immune system [27,32,36,37]. Various studies show that the innate and adaptive immune systems are regulated by hormones, and their control varies depending on the phase of the menstrual cycle [25,38]. During one study, it was found that when PBMCs were infected with HIV-1 sub-subtype A6, v1.A6, high doses of gonadocorticoids induced an increase in mRNA expression of TLR2 and TLR9 genes. At the same time, high doses of estradiol and progesterone increased viral replication (by 3.8 and 4.4 times, respectively), which was consistent with other studies that also showed an increase in VL in the presence of female sex hormones [30,31,33,39]. The mechanisms by which sex steroid hormones enhance HIV-1 replication are not fully understood, but the observed increase in HIV-1 RNA may be partially related to increased TLRs expression. There is evidence that HIV infection is associated with changes in TLRs production, which may contribute to increased HIV-1 replication [40,41]. Thus, several studies have shown an association between elevated TLR2 levels and chronic immune activation and increased viral replication in HIV infection [41,42]. By remodulating cortical actin and creating a more favorable environment for viral gene expression, TLR2 contributes to increased viral production [43]. It has been demonstrated that TLR2 ligands induce HIV-LTR (Long Terminal Repeat) transactivation and HIV-1 replication [44]. It has also been shown ex vivo that the TLR9 ligand, CpG DNA (a short unmethylated CpG dinucleotide), leads to the replication of HIV-1 in the spleen cells of HIV-1 transgenic mice [44]. The increased expression of TLRs in PBMCs infected with the HIV-1 sub-subtype A6 (v1.A6 variant) is consistent with the findings of other authors who have shown that the activation of TLR2 and TLR9 production in HIV-1-infected cells leads to increased virus production [45]. Thus, it can be assumed with a high degree of probability that in this case, one of the factors contributing to the enhancement in virus replication in PBMCs of female donors in the presence of steroid hormones is the gonadocorticoid-induced expression of TLR2 and TLR9 mRNA. TLR2, by facilitating better virus penetration into cells, creates a more favorable environment for the expression of viral genes [43], as does TLR9, which is expressed in host cells and is associated with a higher concentration of HIV-1 RNA [46].

At the same time, when the donor’s PBMCs were infected with subtype B, hormones did not induce any changes in TLRs mRNA expression (with the exception of one donor). The observed difference in TLRs production in cells infected with sub-subtype A6 and subtype B in the presence of hormones can be largely attributed to the high heterogeneity of HIV-1. Currently, there is abundant clinical evidence that there are significant phenotypic differences between HIV-1 subtypes [47,48,49]. Thus, in group M, the genetic variability between HIV-1 subtypes is 25–40% for the env gene and 8–15% for the pol/gag genes [50,51]. There are also significant differences between different subtypes in the regulatory genes vif, nef, and long terminal repeat (LTR) regions. For example, a study of subtypes A and G showed that even minor differences in LTR promoter activity can have a significant impact on the properties of the virus and its replication kinetics [52]. It is known that HIV-1 regulatory proteins are involved in the process of viral replication and help the virus to protect itself from the host immune response [53]. The structural features of these proteins can affect both the progression of HIV infection and the development of the associated diseases [53]. At the same time, it has been shown that the variability of regulatory genes depends on the virus subtype [54]. In particular, the nef sequences of HIV-1 are very diverse. The variations between different subtypes in the nef sequences alone are about 15–24% [55]. For example, studies of the tat and nef proteins of the HIV-1 sub-subtype A6 have revealed specific characteristics that can affect the functional activity of these genes [53,56,57]. Analysis of the Vpu gene sequences of HIV-1 subtype B isolates isolated from patients at different stages of HIV infection revealed a significant difference in their amino acid composition, which may also account for differences in the functional activity of this protein [53]. All these genetic variations inevitably lead to differences in the biological properties of various HIV-1 subtypes.

In addition, a study of the nef gene revealed that in the late stages of HIV infection, there is a phenomenon of splicing in the cell nuclei that is absent in the early stages of infection, which may lead to different phenotypic properties of different isolates of the same HIV-1 subtype [54]. If we take into account that the HIV-1 isolates used in this study were isolated from the blood of patients in the late stages of HIV infection (stages 3 and 4, WHO), then one of the factors that may explain the difference in TLRs expression in the donor’s PBMCs infected with different variants of HIV-1 sub-subtype A6 (v1.A6 and v2.A6) may be the phenomenon of nef regulatory gene splicing.

When donor’s PBMCs were infected with the v1.B variant of subtype B, the exception to the general concept was one donor (Donor 4), in the cells of whom low doses of gonadocorticoids significantly induced an increase in the expression of TLR2 and TLR9, which correlated with an enhanced VL. An increase in the replication of HIV-1 subtype B in the presence of low doses of steroid hormones was also noted by a number of other researchers [37,58]. As in the case of infection with the sub-subtype A6, the v1.A6 variant, it is highly likely that the enhancement in VL is partially due to an increased TLRs expression.

The detected difference in TLRs expression and virus production in the PBMCs of Donor 4 and the PBLs of other donors in the presence of hormones may also be largely due to interhost variability. Thus, numerous studies indicate that a 30% difference in VL in different individuals may be explained by differences in host genetics [59,60,61,62]. Approximately 24.6% of the observed variability in VL can be attributed to genetic polymorphisms in an individual’s gene [59,60]. In particular, immunopolymorphism is an important aspect that determines the susceptibility of the host organism to the pathogen. Polymorphic characteristics in the TLRs encoding gene have been shown to play an important role in inducing differential immune responses of an individual to various infections, influencing the host-pathogen interaction [12,14,63]. Single-nucleotide polymorphism (SPN) of TLRs may be extremely important, given the functional role of many TLRs in HIV infection and the impact of their expression levels on HIV-1 replication [12,14,41,64,65,66,67,68,69,70,71,72]. Thus, the presence of SNP rs111200466 (−196 to −174 Ins/Del) in the TLR2 gene is a risk factor for HIV-1 infection and disease progression [7,73,74]. It has been shown that SNP rs4986790 (1063A/G) and the G allele of SNP rs4986791 in the TLR4 gene are also risk factors for HIV-1 infection and are associated with increased viral load [70,75,76]. There is data that the presence of the TLR9 gene SNP rs352140 (1635A/G) with the GA and GG genotypes is associated with increased viral load and an increased risk of HIV-1 infection, but the mechanisms by which TLR9 affects viral load are still unclear [77].

In this study, we did not detect any changes in TLR4 mRNA production in response to infection with the HIV-1 sub-subtype A6 and subtype B (either with or without the presence of hormones), although several studies have shown that HIV infection can increase TLR4 expression in vivo and in vitro [41,78]. This may be due to the fact that in modulating the expression of this particular TLR, a primary role is played by SNPs which determine the cell’s susceptibility to infection. Additionally, it should be noted that the heterogeneity of the donor pool may also affect the results obtained. It is worth noting that the vast majority of studies on the role of SNPs in the TLR4 gene in HIV infection have been conducted among the Caucasian population of Western Europe. There is almost no data using direct sequencing of patients infected with HIV-1 among representatives of the “Far Eastern race” (a small race within the large Mongoloid race) that prevails in the Russian Far East (in the Amur and Primorsky regions) and among the “Ural population” (the Ural race, which is intermediate between the Mongoloid and Caucasian races), which is widespread in Western and Southern Siberia in Russia. Obviously, further studies are needed to confirm the association of SNPs in the TLR4 gene and vulnerability (susceptibility) to HIV-1 infection in different population groups.

## 5. Conclusions

The results of this study show that gonadocorticoids modulate the expression of TLR2 and TLR9 mRNA in the mononuclear cells of female donors infected with various subtypes of HIV-1 in a different way, and their expression is modulated in a dose-dependent manner. It has been shown that high doses of steroid hormones induce the expression of TLR2 and TLR9 in PBLs infected with sub-subtype A6 (v1.A6), which correlates with an increased VL. It has also been demonstrated that the identified divergences in TLRs expression, which determine the kinetics of viral replication when cells are infected with HIV-1 sub-subtype A6 (v1.A6, and v2.A6 variants), indicate that different isolates of the same subtype have different biological properties. At the same time, it was found that gonadocorticoids did not modulate the expression of TLRs in cells infected with both variants of subtype B, with the exception of one donor. In infected PBMCs of this particular donor, in contrast to the sub-subtype A6, low doses of hormones induced enhanced expression of TLR2 and TLR9. The difference in TLRs expression in cells from different donors infected with the HIV-1 subtype B (v1.B) in the presence of hormones suggests that individual genetic factors also play a significant role in the susceptibility of PBLs to HIV infection and viral replication.

Thus, the data obtained indicate that gonadal steroids can have a significant impact on HIV-1 replication by modulating pattern recognition receptors (PRRs), such as TLRs, depending on the HIV-1 subtype and host genetics.

There are certain limitations of this work. We specifically refused to conduct this research exclusively on the macrophage lineage with the intention of bringing the experimental conditions as close as possible to the in vivo conditions, since in the human body, all cells of the immune system participate to one degree or another in the response to the pathogen. However, there is evidence that the regulation of TLRs pathways differs somewhat depending on the type of cells involved in the immune response [8,79]. Given this fact, in future experiments, we plan to take into account the differentiation of monocytes, myeloid, and plasmacytoid dendritic cells. This will help us to better understand the relationship between the modulation of TLRs expression by gonadal steroids and the replication of HIV-1.

## Data Availability

The original contributions presented in this study are included in the article/Supplementary Material. Further inquiries can be directed to the corresponding author.

## Supplementary Materials

The following supporting information can be downloaded at: https://www.mdpi.com/article/10.3390/v17111512/s1, Table S1. HIV-1 isolates Resistance Summary, Table S2. HIV-1 isolates tropism, Figure S1. Median values of TLR4 expression in PBLs of female donors infected with HIV-1 sub-subtype A6 and subtype B in the presence of (L) low and (H) high doses of estradiol (Estr) and progesterone (Prog), Figure S2. Median levels of CCR5 and CCR8 co-receptors expression in PBMCs infected with HIV-1 sub-subtype A6 and subtype B in the presence of high doses of gonadal steroids.

## Abbreviations

The following abbreviations are used in this manuscript:

PBMCs	Human peripheral blood mononuclear cells
TLRs	Toll-like receptors
Estr	Estradiol
Prog	Progesterone
SNP	Single-nucleotide polymorphism
