# Supplementary material for: Gonadocorticoids Have Different Effects on the Expression of Toll-like Receptors When Infected with Various HIV-1 Subtypes
Source: Viruses. 2025 Nov 18;17(11):1512. doi: 10.3390/v17111512 (PMC12656866; doi:10.3390/v17111512)
Supplement: Supplementary file 1 [file viruses-17-01512-s001.zip › Supplementary File Figure S1.pdf]

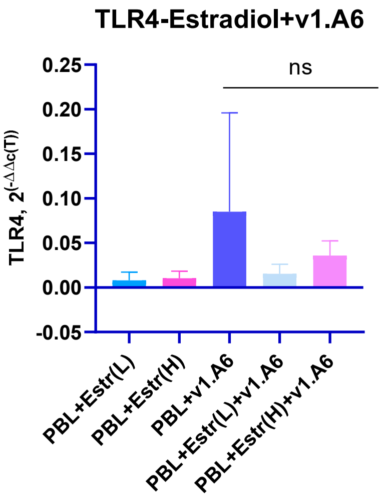
TLR4-Estradiol+v1.A6
TLR4, 2^(-ΔΔc(T))
0.25
0.20
0.15
0.10
0.05
0.00
-0.05
ns
PBL+Estr(L)
PBL+Estr(H)
PBL+v1.A6
PBL+Estr(L)+v1.A6
PBL+Estr(H)+v1.A6

A

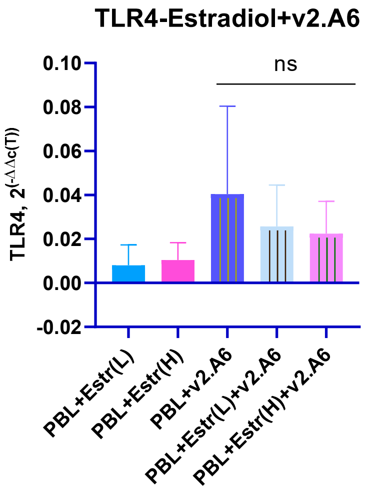
TLR4-Estradiol+v2.A6
TLR4, 2^(-ΔΔc(T))
0.10
0.08
0.06
0.04
0.02
0.00
-0.02
ns
PBL+Estr(L)
PBL+Estr(H)
PBL+v2.A6
PBL+Estr(L)+v2.A6
PBL+Estr(H)+v2.A6

B

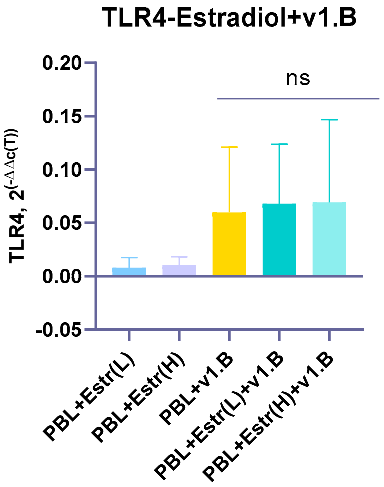
TLR4-Estradiol+v1.B
TLR4, 2^(-ΔΔc(T))
0.20
0.15
0.10
0.05
0.00
-0.05
ns
PBL+Estr(L)
PBL+Estr(H)
PBL+v1.B
PBL+Estr(L)+v1.B
PBL+Estr(H)+v1.B

C

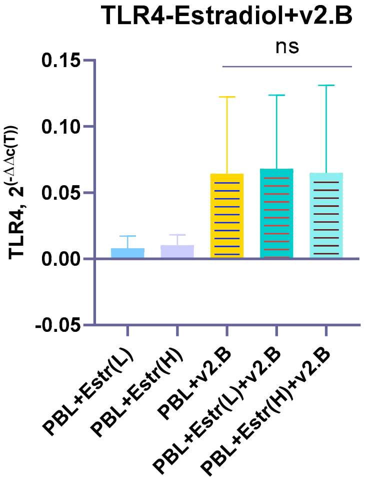
TLR4-Estradiol+v2.B
TLR4, 2^(-ΔΔc(T))
0.15
0.10
0.05
0.00
-0.05
ns
PBL+Estr(L)
PBL+Estr(H)
PBL+v2.B
PBL+Estr(L)+v2.B
PBL+Estr(H)+v2.B

D

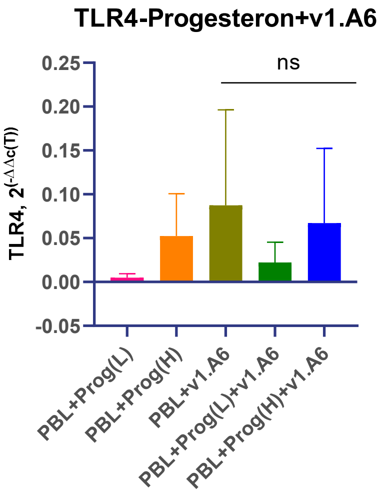
TLR4-Progesteron+v1.A6
TLR4, 2^(-ΔΔc(T))
0.25
0.20
0.15
0.10
0.05
0.00
-0.05
ns
PBL+Prog(L)
PBL+Prog(H)
PBL+v1.A6
PBL+Prog(L)+v1.A6
PBL+Prog(H)+v1.A6

E

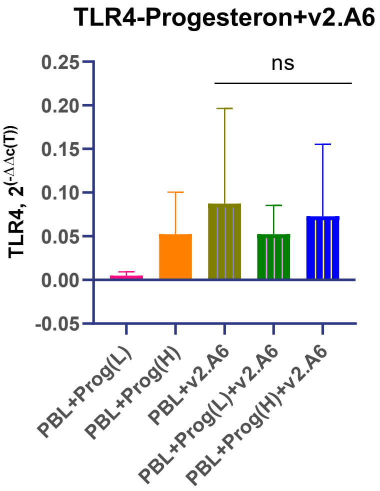
TLR4-Progesteron+v2.A6
TLR4, 2^(-ΔΔc(T))
0.25
0.20
0.15
0.10
0.05
0.00
-0.05
ns
PBL+Prog(L)
PBL+Prog(H)
PBL+v2.A6
PBL+Prog(L)+v2.A6
PBL+Prog(H)+v2.A6

F

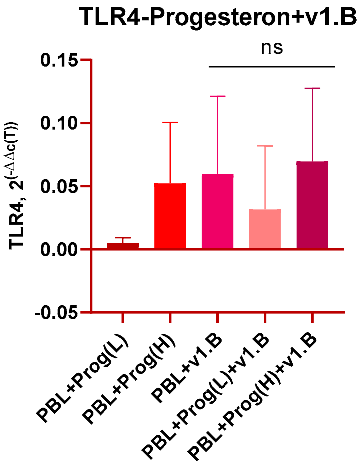

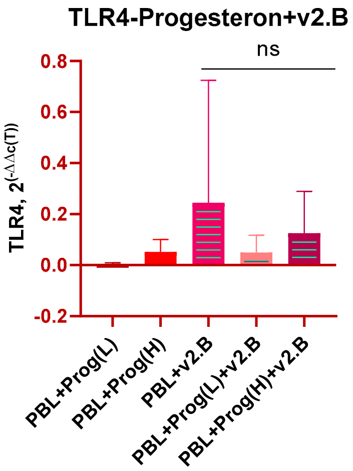

G H

Supplementary FigureS1. Median values of TLR4 expression in PBLs of female donors infected with HIV-1 sub-subtype A6 and subtype B in the presence of (L) low ( ) and (H) high doses of estradiol (Estr) and progesterone (Prog): (A) PBLs+A6, v1.A6+Estradiol ; (B) PBLs+A6, v2.A6+Estradiol; (C) PBLs+B, v1.B+Estradiol; (D) PBLs+B, v2.B+Estradiol; (E) PBLs+A6, v1.A6+Progesterone ; (F) PBLs+A6, v2.A6+Progesterone; (G) PBLs+B, v1.B+Progesterone; (H) PBLs+B, v2.B+Progesterone. All data points are the average of three culture wells run in triplicate. ns, no statistical difference, $p > 0.05$. p-values were generated by one-way ANOVA with multiple comparisons. The median values of each of the three experiments were used to present the statistical analysis (n=3).
