# Supplementary material for: Gonadocorticoids Have Different Effects on the Expression of Toll-like Receptors When Infected with Various HIV-1 Subtypes
Source: Viruses. 2025 Nov 18;17(11):1512. doi: 10.3390/v17111512 (PMC12656866; doi:10.3390/v17111512)
Supplement: Supplementary file 1 [file viruses-17-01512-s001.zip › Supplementary File Figure S2.pdf]

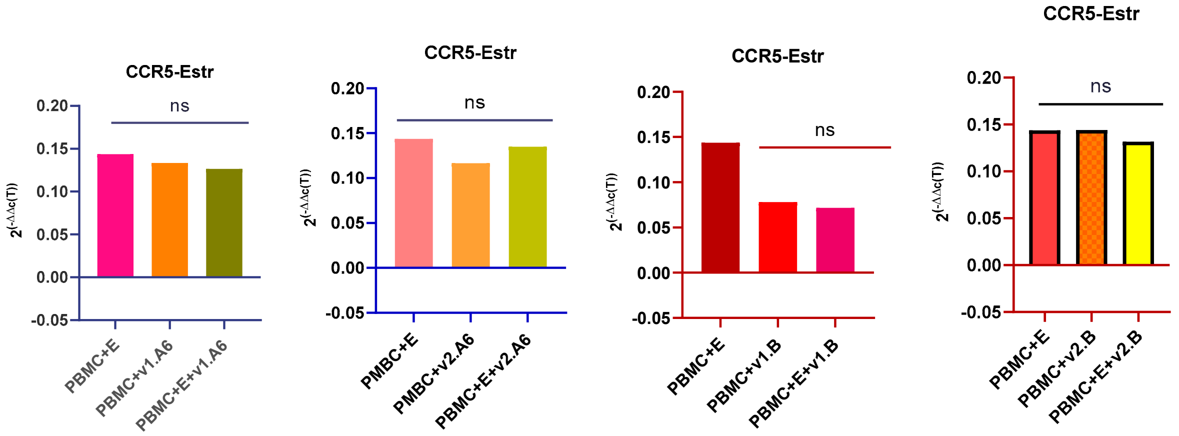

(A) (B) (C) (D)

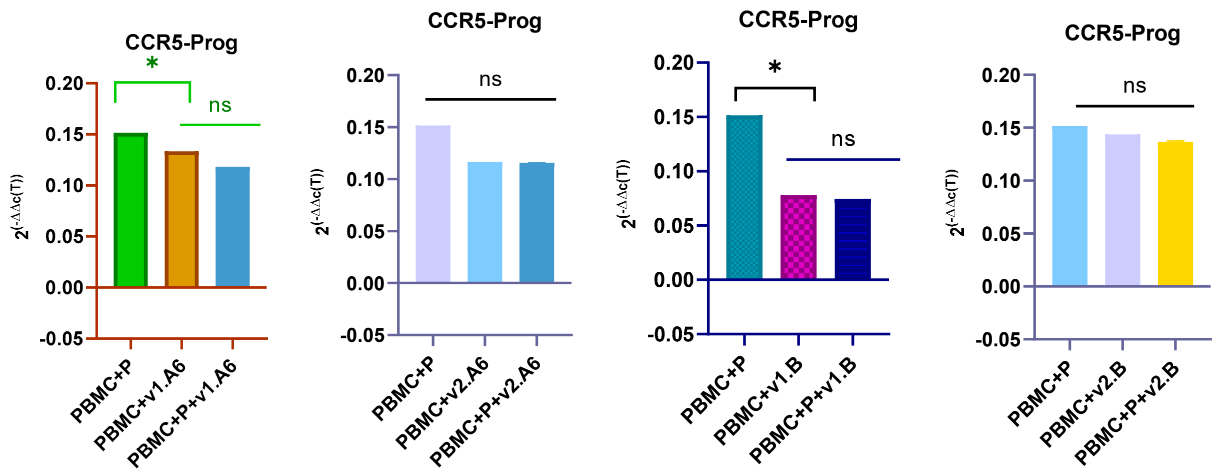

E F G H

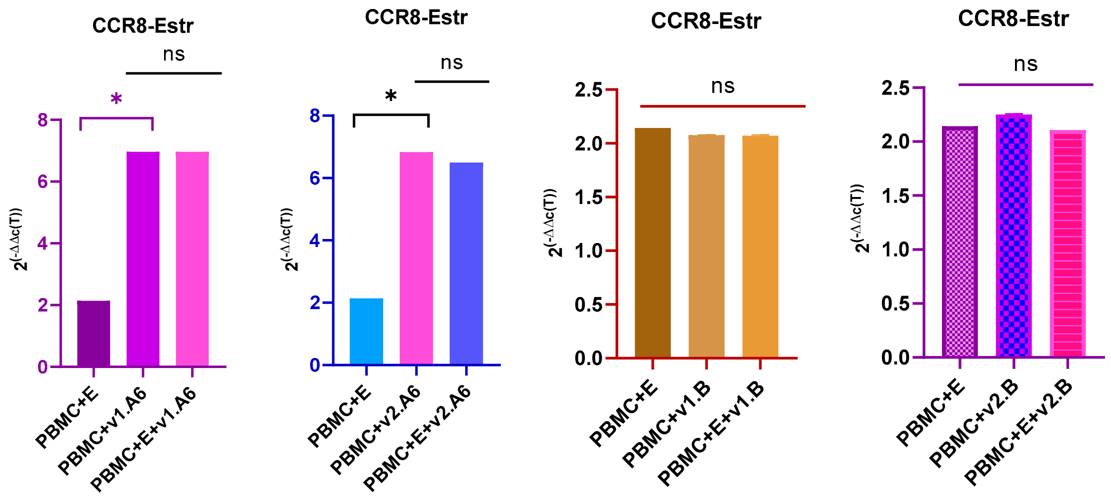

I J K L

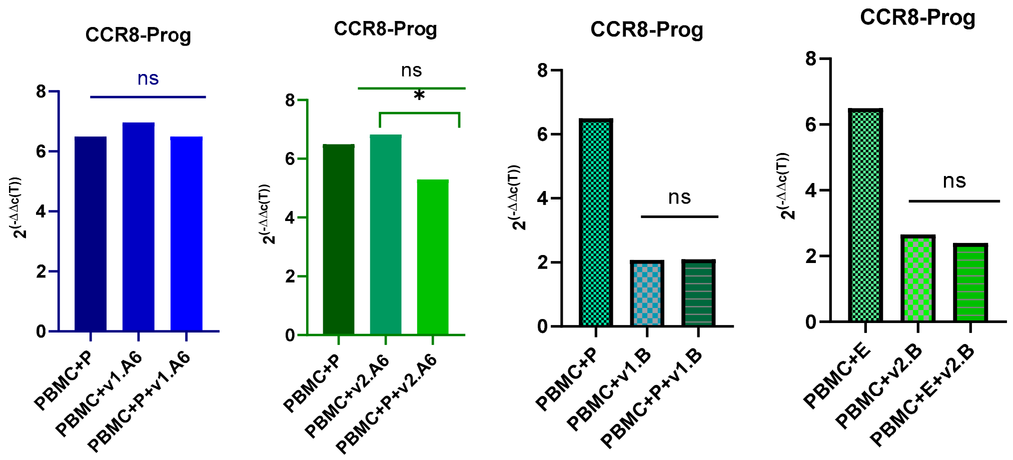

M N O P

Supplementary FigureS2. Median levels of CCR5 and CCR8 co-receptors expression in PBMCs infected with HIV-1 sub-subtype A6 and subtype B in the presence of high doses of gonadal steroids. PBMC- peripheral blood mononuclear cells; E- estradiol; P – progesterone; v1.A6 - sub-subtype A6 variant 1; v2.A6 - sub-subtype A6 variant 2; v1.B – subtype B variant 1; v2.B – subtype B variant 2; (A) CCR5- v1.A6 + Estradiol; (B) CCR5 -v2.A6 + Estradiol; (C) CCR5- v1.B + Estradiol; (D) CCR5- v2.B + Estradiol; (E) CCR5 - v1.A6 + Progesterone; (F) CCR5- v2.A6 + Progesterone; (G) CCR5 -v1.B + Progesterone; (H) CCR5 -v2.B + Progesterone; (I) CCR8- v1.A6 + Estradiol; (J) CCR8 - v2.A6 + Estradiol; (K) CCR8- v1.B + Estradiol; (L) CCR8 - v2.B + Estradiol; (M) CCR8 - v1.A6 + Progesterone; (N) CCR8 - v2.A6 + Progesterone; (O) CCR8 - v1.B + Progesterone; (P) CCR8 - v2.B + Progesterone; ns, no statistical difference; * $p < 0.05$. The median values of each of the three experiments were used to present the statistical analysis (n=3).
